# Topical Tacrolimus as an adjunct to Conventional Therapy for Stromal Herpetic Keratitis: a Randomized Clinical Trial

**DOI:** 10.18502/jovr.v14i4.5437

**Published:** 2019-10-24

**Authors:** Mitra Akbari, Reza Soltani Moghadam, Ramin Elmi, Amir Nosrati, Ehsan Taghiabadi, Nasser Aghdami

**Affiliations:** ^1^Eye Research Center, Amiralmomenin Hospital, School of Medicine, Guilan University of Medical Sciences, Rasht, Iran; ^2^Legal Medicine Organization, Rasht, Iran; ^3^Department of Regenerative Biomedicine at Cell Science Research Center, Royan Institute for Stem Cell Biology and Technology, ACECR, Tehran, Iran

**Keywords:** Corneal Haziness, Corneal Neovascularization, Corneal Sequel, Herpes Simplex Keratitis, Herpetic Stromal Keratitis, Tacrolimus

## Abstract

**Purpose:**

This study investigates the effects of 0.05% topical tacrolimus as an adjunct therapy for patients with non-necrotizing herpetic stromal keratitis (HSK).

**Methods:**

Patients with non-necrotizing HSK, referred to the Cornea Clinic at Hospital in Rasht, Iran, between September 2016 and February 2018, were randomly assigned to two groups. The case group (*N* = 25) and the control group (*N* = 25) received conventional treatment with systemic acyclovir and topical prednisolone. The case group (*N* = 25) additionally received 0.05% tacrolimus eye drops four times a day for one month. Complete ocular examinations, including best-corrected visual acuity (BCVA) assessment, intraocular pressure (IOP) measurement, slit lamp biomicroscopy, and photo slit lamp imaging, were performed before treatment, and 3, 7, 14, 21, and 28 days after the intervention.

**Results:**

The mean age of the patients was 46.2 ± 12.9 years, and 70% of the patients were male. There was no difference between the groups in terms of age, sex, and baseline ocular measurements (*P* > 0.05). The case group had a lower mean logarithm of the minimum angle of resolution (LogMAR) for BCVA, lower grading scores, and steeper decreasing trends for corneal haziness, edema, neovascularization, and epitheliopathy compared to the control group after the second week (*P* < 0.05), while IOP remained unchanged between groups (*P* > 0.05).

**Conclusion:**

The addition of 0.05% topical tacrolimus enhances visual acuity and reduces corneal inflammation, neovascularization, and scarring; thus, it can used as an appropriate adjunct treatment for patients with HSK.

##  INTRODUCTION

Herpes simplex virus type 1 (HSV-1) is a highly infectious virus, with more than half of the general population being seropositive for the virus in many countries.^[1,2]^ HSV-1 not only causes recurrent oral lesions but is also associated with several extra-oral manifestations, such as cutaneous and ocular complications.^[3,4]^ The eye complications may present as blepharitis, conjunctivitis, corneal epithelial keratitis, and herpetic stromal keratitis (HSK), which is the leading cause of infectious blindness in developed countries and can cause irreversible corneal scarring and thinning, neovascularization, lipid keratopathy, and eventual blindness after several recurrent episodes. ^[5,6]^ HSK is immunomediated and occurs as a result of chronic viral reactivation.^[5]^ Subsequent episodes lead to angiogenesis and neovascularization which can have a negative impact on corneal transplantation that may be needed in these patients.

With an estimated incidence rate of 23.3% and increasing prevalence in developed nations,^[7]^ HSK is treated using antiviral therapies, such as acyclovir, and topical corticosteroids. However, even combination of these treatments can create challenges and adverse effects,^[8,9]^ and researchers continue to seek new treatments.^[10]^ Considering the immune-mediated pathogenesis of corneal scarring and neovascularization in HSK, immune regulatory drugs, such as cyclosporine A, have been introduced as attractive alternatives for the management of the disease.^[11,12]^

Tacrolimus is an immunosuppressive macrolide that is 100 times more effective than cyclosporine A at suppressing both B-cell and T-cell activation, T helper responses, and the production of interleukins.^[13]^ Considering the possible role of cell-mediated (CD4${}^{+}$ and CD8${}^{+}$) response, interleukins, and cytokines in the pathophysiology of stromal keratitis in HSK,^[4][5,6][7][8,9][10][11,12][13][14][15][16]^ tacrolimus has been suggested as an effective treatment for HSK and corneal neovascularization.^[17,18]^ Tacrolimus has also been found to be effective for treating different types of keratitis including vernal and atopic keratoconjunctivitis.^[19,20]^ The present study was performed to investigate the effects of 0.05% topical tacrolimus on HSK compared to a control group.

##  METHODS

###  Study design

This randomized clinical trial (RCT) was performed on patients with non-necrotizing HSK. The samples were selected from patients referred to the Emergency Department and Cornea Clinic of Hospital in Rasht, Iran, between September 2016 and February 2018. The study began after the approval of the study protocol by the Ethics Committee of Guilan University of Medical Sciences and the Registry of Clinical Trials (IRCT Id: IRCT2016102429871N2). Fifty patients were assessed after excluding the patients who were lost to be followed-up.

Only patients who were diagnosed with active HSK, which is primarily diagnosed by its clinical presentation in slit lamp examinations were included in this study. Patients with other types of keratitis were eliminated upon reviewing medical histories, evaluations, and laboratory tests. In suspected cases, tear samples, aqueous tap, and corneal scrapings were analyzed using polymerase chain reaction (PCR) for identification of HSV-1. PCR was used for HSV-1 identification only in two suspected HSK patients. Patients with a positive history of corneal surgery or ocular trauma, patients who were pregnant or lactating during the study period, patients with necrotizing HSK, and patients who were under treatment with any systemic or topical immunosuppressive drugs concurrently were also excluded. We only selected patients who had not used systemic acyclovir and topical steroids prior to their inclusion in this study.

After patient selection, the design and objectives of the study was explained to the participants who signed a written informed consent form that clarified the groups and interventions, possible adverse effects of each therapeutic protocol, and the patients' freedom to leave the study whenever they wished. All ethical considerations were met throughout the study according to the Helsinki's Declaration. The included patients were randomly assigned to two equal groups based on a block randomization (i.e., 4 patients per block). The control group comprised of 25 patients who received treatments with 400 mg acyclovir tablets (Aciclovir 400, Darou Pakhsh, Tehran, Iran) either two times a day for interstitial keratitis or five times a day if accompanied by iridocyclitis for 14 days, and 1% topical prednisolone eye drop (Precord 1%, Sina Darou, Tehran, Iran) every two hours with a two-hour dose reduction every week. For the case group consisting of 25 patients, in addition to the conventional treatment with oral acyclovir and topical prednisolone (as described earlier), 0.05% tacrolimus eye drops were prescribed four times a day for one month.

Before the intervention and on days 3, 7, 14, 21, and 28 after treatment, best corrected visual acuity (BCVA), intraocular pressure (IOP), and a complete slit lamp examination were performed and photo slit lamp findings were documented using digital corneal photographs (Imagenet, Topcon SL-8Z, Tokyo, Japan). Scores, ranging from 0–3, were assigned to HSK corneal parameters (corneal haziness, edema, vascularization, and the results of fluorescence staining of punctate epitheliopathy) according to Table 1. These scores were determined by two masked observers (cornea specialists). Since there was no prior approved or validated method for this assessment, we defined scoring system. The total grading scores were recorded at each examination, and the results were evaluated before and after the treatment of each patient at follow-up visits. In this study, the primary outcome measure was the change in the corneal haziness and edema score and the secondary outcome measure was the change in corneal vascularization and epitheliopathy score These outcomes were compared between the groups. Patients were questioned about potential side effects caused by tacrolimus eye drops at each visit, and the results were recorded in a checklist.

**Table 1:** The grading scores for the corneal parameters in herpetic stromal keratitis (HSK)


**Corneal parameters scoring in herpetic stromal keratitis (HSK)**	**Score 0**	**Score 1**	**Score 2**	**Score 3**
**Corneal vascularization**	No evidence	1 quadrant	2 quadrants	≥ 3 quadrants
Corneal punctate* epitheliopathy	None	Mild	Moderate	Severe
Corneal haziness**	None	Mild	Moderate	Severe
Corneal edema ***	None	Mild	Moderate	Severe
	
	
*Mild: Scattered fluorescein staining; Moderate: Confluent fluorescein staining; Severe: Compact fluorescein staining				
**Mild corneal haze: Iris details clearly visible; Moderate corneal haze: Iris details not clearly visible; Severe corneal haze/opacification: Anterior chamber structures not visible				
***Mild edema: Minimal loss of transparency; Moderate edema: Dull glass, edema does not extend past the anterior half of the stroma; Severe edema: Involvement of the entire thickness of the stroma				

###  Tacrolimus eye drop preparation

Tacrolimus eye drops were reconstituted by adding a balanced salt solution to a tacrolimus vial (Prograf, Astellas Pharma Inc., Dublin, Ireland) under sterile conditions to achieve a 0.05% concentration of tacrolimus. After the preparation, drops were stored in a refrigerated room at 4°C.

###  Statistical analysis

Results were presented as the mean ± standard deviation (SD) for quantitative variables and were summarized by frequency (percentage) for categorical variables. First, Kolmogorov–Smirnov tests were conducted to assess the normal distribution of data, and variables with normal distribution were compared using independent *T*-test or one way analysis of variance (ANOVA), and the variables without a normal distribution were compared using Mann–Whitney and Kruskal–Wallis tests. Categorical variables were compared using the chi-square test. The values at each interval were also compared to the baseline value in each group, and the results were reported as mean changes. For the statistical analysis, the statistical software IBM SPSS Statistics for Windows version 21.0 was used, and *P* ≤ 0.05 was considered statistically significant. Power analysis was performed with the PASS sample size software.

##  RESULTS

Data analysis was performed on 50 eyes of 50 subjects in two equal groups; 72 and 68% of the control group and the case group were male, respectively; *P* = 0.76). The PCR test was used for HSV-1 identification in only two suspected HSK patients. One of these two patients was excluded from the study due to the negative test result and the lack of a definitive diagnosis.

**Figure 1:**
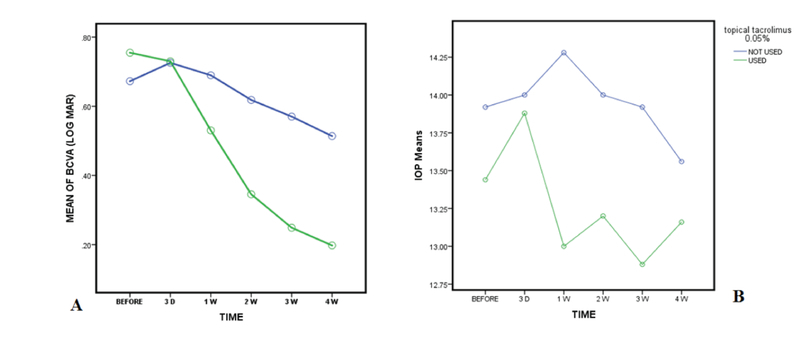
The trend of mean LogMAR of best corrected visual acuity (BCVA) (A) and intraocular pressure (IOP) (B) at baseline, 3 days, 1, 2, 3, and 4 weeks after the intervention in the case (used tacrolimus) and the control (tacrolimus was not used) groups.

The mean ± SD of the patients' age was 46.2 ± 12.9 (range: 24–81) years. There was no significant difference regarding age between the two groups (control group: 46.0 ± 13.32 years; case group: 46.4 ± 12.79 years; *P* = 0.91). The comparison of the logarithm of the minimum angle of resolution (LogMAR) for BCVA between the groups showed significant differences after the end of the second week of the interventions. The mean, median, and 25th and 75th percentiles for the LogMAR of the BCVA on days 14, 21, and 28 after the intervention were smaller for the case group compared to the control group (*P* < 0.001) [Table 2]. Trends in changes in the LogMAR of the BCVA are shown in Figure 1(A). The case group had a steeper decrease in LogMAR of BCVA than the control group.

**Table 2:** Comparison of the best corrected visual acuity and intraocular pressure at the baseline, 3, 7, 14, 21, and 28 days after the intervention between the groups


	**Corrected visual acuity (Log)**			**Intraocular pressure**		
	**Control group**	**Case group**	P-value*	**Control group**	**Case group**	**P-value**
**Before intervention**	Mean ± Standard Deviation	0.67 ± 0.38	0.75 ± 0.35	0.425	13.92 ± 3.68	13.44 ± 2.97	0.882
	Median	0.7	0.7	14	13	
	percentile	0.3	0.52	1	11	
	percentile	1	1	15	15	
**Day 3 after intervention**	Mean ± Standard Deviation	0.73 ± 0.37	0.73 ± 0.32	0.921	14.00 ± 3.06	13.88 ± 3.05	0.93
	Median	0.7	0.7	14	14	
	25^th^ percentile	0.4	0.52	12	12	
	75^th^ percentile	1	1	15	15	
	Mean Rank	24.48	26.52	0.479	26.44	24.56	0.626
**Day 7 after intervention**	Mean ± Standard Deviation	0.69 ± 0.36	0.53 ± 0.30	0.117	14.28 ± 3.36	13.00 ± 2.68	0.206
	Median	0.7	0.52	14	13	
	25^th^ percentile	0.4	0.3	12	11	
	75^th^ percentile	1	0.7	17	15	
	Mean Rank	16.46	34.54	< 0.001	23.46	27.54	0.308
**Day 14 after intervention**	Mean ± Standard Deviation	0.62 ± 0.32	0.35 ± 0.20	< 0.001	14.00 ± 3.23	13.20 ± 2.84	0.426
	Median	0.52	0.3	14	14	
	25^th^ percentile	0.4	0.22	11	1	
	75^th^ percentile	1	0.4	15	15	
	Mean Rank	15.74	35.26	< 0.001	24.88	26.12	0.754
**Day 21 after intervention**	Mean ± Standard Deviation	0.57 ± 0.29	0.25 ± 0.21	< 0.001	13.92 ± 3.13	12.88 ± 2.73	0.236
	Median	0.52	0.22	14	13	
	25^th^ percentile	0.3	0.15	11	1	
	75^th^ percentile	0.7	0.3	15	15	
	Mean Rank	15.6	35.4	< 0.001	23.26	27.74	0.257
**Day 28 after intervention**	Mean ± Standard Deviation	0.51 ± 0.28	0.20 ± 0.20	< 0.001	13.56 ± 2.81	13.16 ± 2.61	0.601
	Median	0.52	0.15	14	14	
	25^th^ percentile	0.22	0.1	11	11	
	75^th^ percentile	0.7	0.22	15	15	
	Mean Rank	16.8	34.2	< 0.001	24.8	26.2	0.723
	
	
*The results of the chi-square test are reported at a significance level of 0.05							

There were no significant differences in the mean, median, and the 25th and 75th percentiles between the groups, and no significant difference in the mean changes in IOP at each interval after the intervention compared to baseline (*P* > 0.05). The results of IOP measurements are shown in Table 2, and Figure 1(B) shows that the changes in the IOP were not consistent in the case group, while the control group had a more consistent decrease in IOP after seven days following the intervention.

**Figure 2:**
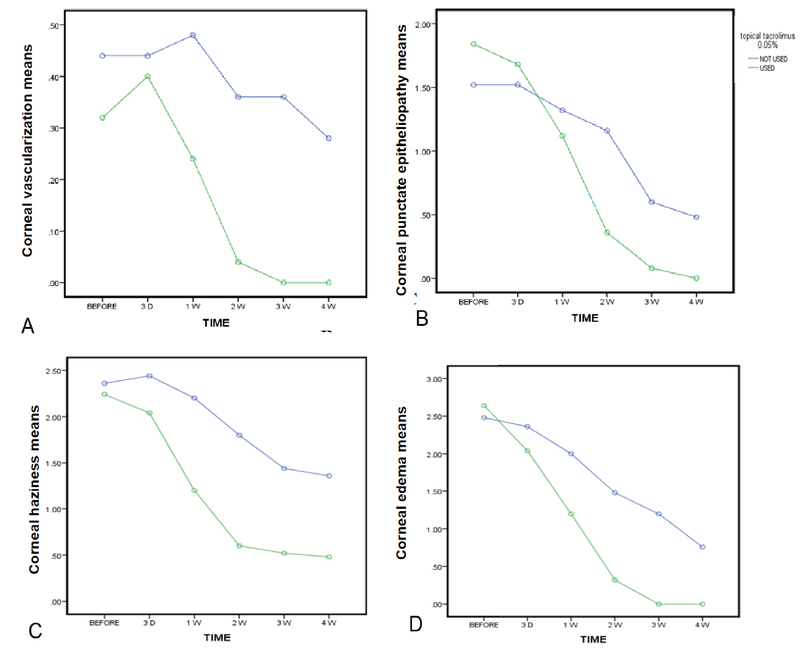
The trend of decrease in mean corneal vascularization (A), punctuate epitheliopathy (B), haziness (C), and edema (D) scores at baseline, 3 days, 1, 2, 3, and 4 weeks after the intervention in the case (used tacrolimus) and the control (tacrolimus was not used) groups.

**Figure 3:**
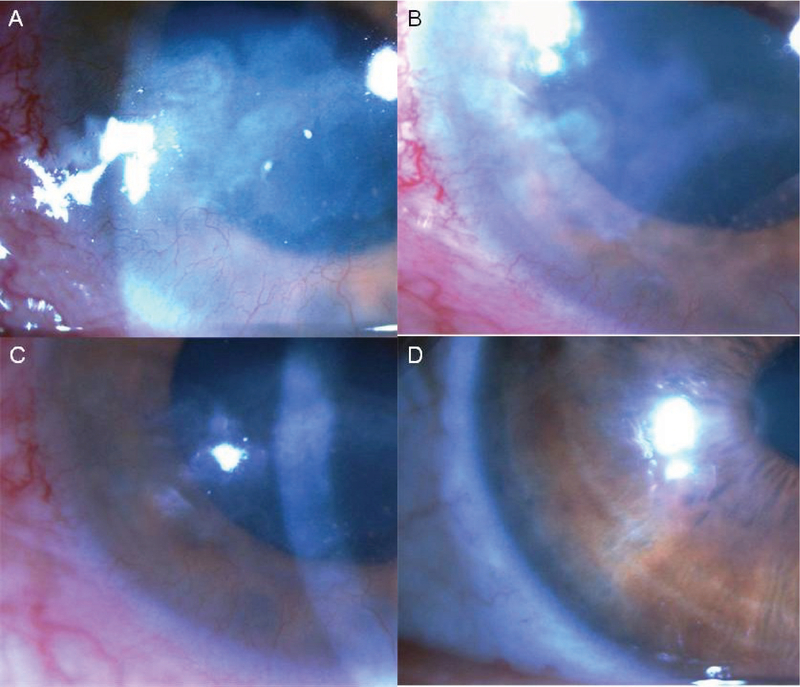
The significant decrease in corneal vascularization and haziness scores after addition of topical tacrolimus eye drops 0.05% at baseline (A), and after 1 week (B), 2 weeks (C), and 4 weeks (D).

Mean corneal vascularization grading scores between the groups were significantly different. The mean corneal vascularization grading scores were lower in the case group than the control group on days 14 (*P *= 0.005), 21 (*P* = 0.001), and 28 (*P* = 0.005) after the intervention [Table 3]. There were no significant differences in mean changes between the groups (*P* > 0.05) [Table 3]. As shown in Figure 2(A), the corneal vascularization scores decreased in both groups but the decrease was steeper in the case group compared to the control group [Figure 3].

**Table 3:** Comparison of the corneal vascularization at the baseline, 3, 7, 14, 21, and 28 days after the intervention between the groups


**Intervals**	**Classification**	**Corneal neovascularization, No.(%)**		
	**Control group**	**Case group**	**P-value**
**Before intervention**	Not vascularized	16(64)	18(72)	0.51
	1 quadrant	7(28)	6(24)	
	2 quadrants	2(8)	1(4)	
	≥ 3 quadrants	0(0)	0(0)	
	Total mean grade	26.6	24.4	
**Day 3 after intervention**	Not vascularized	16(64)	16(64)	0.92
	1 quadrant	7(28)	8(32)	
	2 quadrants	2(8)	1(4)	
	≥ 3 quadrants	0(0)	0(0)	
	Total mean grade	25.68	25.32	
	Mean Rank	26.5	24.5	0.15
**Day 7 after intervention**	Not vascularized	15(60)	19(76)	0.18
	1 quadrant	8(32)	6(24)	
	2 quadrants	2(8)	0(0)	
	≥ 3 quadrants	0(0)	0(0)	
	Total mean grade	27.74	23.26	
	Mean Rank	24.08	26.92	0.25
**Day 14 after intervention**	Not vascularized	16(64)	24(96)	0.005
	1 quadrant	9(36)	1(4)	
	2 quadrants	0(0)	0(0)	
	≥ 3 quadrants	0(0)	0(0)	
	Total mean grade	29.5	21.5	
	Mean Rank	23.62	27.38	0.25
**Day 21 after intervention**	Not vascularized	16(64)	25(100)	0.001
	1 quadrant	9(36)	0(0)	
	2 quadrants	0(0)	0(0)	
	≥ 3 quadrants	0(0)	0(0)	
	Total mean grade	3	21	
	Mean Rank	23.2	27.8	0.145
**Day 28 after intervention**	Not vascularized	18(72)	25(100)	0.005
	1 quadrant	7(28)	0(0)	
	2 quadrants	0(0)	0(0)	
	≥ 3 quadrants	0(0)	0(0)	
	Total mean grade	29	22	
	Mean Rank	24.16	26.84	0.418
	
	
*The results of the chi-square test are reported at a significance level of 0.05				

The mean corneal punctate epitheliopathy scores were significantly different between the groups, with the scores being significantly lower in the case group than in the control group on days 21 and 28 after the intervention. There were significant changes in the mean corneal punctate epitheliopathy scores in the groups on days 14, 21, and 28 after the intervention (*P* < 0.01). The changes in the corneal punctate epitheliopathy scores are shown in Figure 2(B). These mean changes were steeper in the case group than the control group.

**Figure 4:**
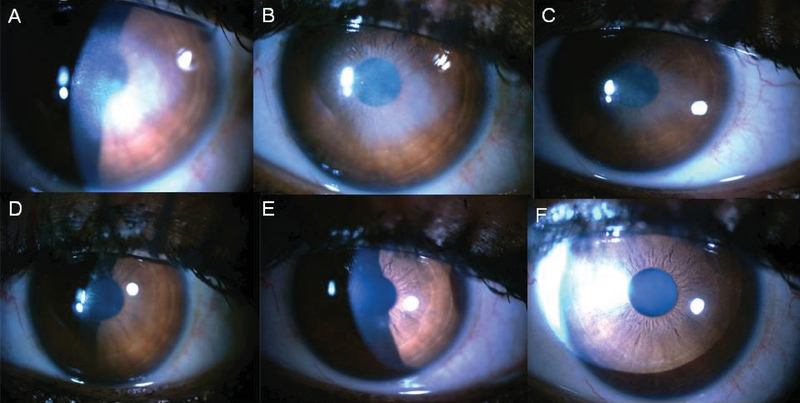
The significant decrease in corneal haziness and edema scores after addition of topical tacrolimus eye drops 0.05% at baseline (A), and after 3 days (B), 1 week (C), 2 weeks (D), 3 weeks (E), and 4 weeks (F).

The mean corneal haziness scores were significantly lower in the case group than the control group on days 7, 14, 21, and 28 (all *P*s = 0.001). The mean changes in the scores were significantly different between the groups, with the case group exhibiting significantly higher mean changes at all intervals (*P* < 0.01) [Table 4]. Figure 2(C) shows a decreasing trend in corneal haziness for both groups. However, the decrease in corneal haziness was steeper for the case group compared to the control group [Figure 4].

**Table 4:** Comparison of the corneal haziness and edema at the baseline, 3, 7, 14, 21, and 28 days after the intervention between the groups


**Intervals**	**Corneal haziness, No. (%)**			**Corneal edema, No. (%)**		
	**Classification**	**Control group**	**Case group**	P-value*	**Control group**	**Case group**	P-value*
**Before intervention**	No evidence	0(0)	0(0)	0.52	0(0)	0(0)	0.34
	Mild	2(8)	3(12)	1(4)	0(0)	
	Moderate	12(48)	13(52)	11(44)	9(36)	
	Severe	11(44)	9(36)	13(52)	16(64)	
	Mean grade	26.7	24.3	23.82	27.18	
**Day 3 after intervention**	No evidence	0(0)	0(0)	0.07	0(0)	0(0)	0.05
	Mild	1(4)	7(28)	3(12)	3(12)	
	Moderate	12(48)	10(40)	10(40)	18(72)	
	Severe	12(48)	8(32)	12(48)	4(16)	
	Mean grade	28.98	22.02	29.02	21.98	
	Mean rank	22.2	28.8	0.01	19.8	31.2	0.001
**Day 7 after intervention**	No evidence	0(0)	4(16)	0.001	0(0)	2(8)	0.001
	Mild	5(20)	13(52)	9(36)	16(64)	
	Moderate	10(40)	7(28)	7(28)	7(28)	
	Severe	10(40)	1(4)	9(36)	0(0)	
	Mean grade	33.1	17.9	31.26	19.38	
	Mean rank	16.56	34.44	0.001	16.58	34.42	0.001
**Day 14 after intervention**	No evidence	0(0)	12(48)	0.001	1(4)	17(68)	0.001
	Mild	8(32)	11(44)	13(52)	8(32)	
	Moderate	14(56)	2(8)	9(36)	0(0)	
	Severe	3(12)	0(0)	2(8)	0(0)	
	Mean grade	35.04	15.96	35.26	15.74	
	Mean rank	15.7	35.3	0.001	14.02	36.98	0.001
**Day 21 after intervention**	No evidence	0(0)	13(52)	0.001	2(8)	25(100)	0.001
	Mild	14(56)	11(44)	16(64)	0(0)	
	Moderate	11(44)	1(4)	7(28)	0(0)	
	Severe	0(0)	0(0)	0(0)	0(0)	
	Mean grade	34.14	16.86	37	14	
	Mean rank	17	34	0.001	14.44	36.56	0.001
**Day 28 after intervention**	No evidence	0(0)	14(56)	0.001	7(28)	25(100)	0.001
	Mild	16(64)	10(40)	17(68)	0(0)	
	Moderate	9(36)	1(4)	1(4)	0(0)	
	Severe	0(0)	0(0)	0(0)	0(0)	
	Mean grade	33.98	17.02	34.5	16.5	
	Mean rank	17.52	33.48	0.001	16.56	34.44	0.001
	
	
*The results of chi-square test are reported at a significance level of 0.05.							

The mean corneal edema scores were significantly lower in the case group than in the control group on days 7, 14, 21, and 28 (all *P*s = 0.001). The mean changes were significantly higher in the case group at all intervals (all *P*s = 0.001) [Table 4]. Figure 2(D) shows the decreasing trend of corneal edema in both groups. This decrease was steeper in the case group than the control group. Patients who received topical tacrolimus did not report any discomfort or complications and all patients tolerated the topical tacrolimus well.

##  DISCUSSION 

In this prospective study involving 50 HSK patients, the case and control groups were matched in terms of age, sex, and all baseline ocular measurements to allow comparison without confounders. The case group received 0.05% tacrolimus eye drops in addition to the conventional treatment, and the control group received only the conventional treatment. Ocular measurements were performed at six intervals and were compared between the groups.

The results showed that both groups exhibited improved BCVA with a decreasing trend in the LogMAR of BCVA. However, the trend in the case group was very steep, with a mean change of 34.20 after one month; while that in the control group was gentle, with a mean change of 16.80. There were significant differences between the groups on days 7, 14, 21, and 28 after the intervention. These results indicate that the addition of tacrolimus enhances visual improvement in cases with HSK. The final visual acuity of the case group was superior to that of the control group. Cyclosporine A was also found to be effective in improving visual acuity in HSK cases that were resistant to conventional therapy.^[12]^ There are no previous tacrolimus studies in patients with HSK to which the results of the present study can be compared.

Corneal vascularization was measured in both groups at six intervals. A decreasing trend was observed in both groups. However, the reduction trend was significantly steeper in the case group compared to the control group. A lower mean grading score for neovascularization was observed in the case group on days 14, 21, and 28 after the intervention. As shown in previous studies, the anti-angiogenic effect of tacrolimus can be comparable to that of bevacizumab.^[18]^ As we observed in the present trial, topical tacrolimus was effective in reducing corneal neovascularization during stromal HSK and can potentially reduce the risk for future keratoplasty and corneal transplantation in these cases.

The mean changes in corneal edema and haziness were higher seven days after treatment in the case group, indicating that both treatments reduced corneal vascularization, edema, and haziness grades. However, this reduction was significantly higher in the case group. Consistent with these results, Eric et al also reported reduced corneal vascularization and edema with significant decreases in groups receiving 0.03% or 0.1% tacrolimus after 14 days, compared to a control group.^[17]^ Neovascularization and haziness are important indicators of the progression of ocular lesions and the main determinants of corneal blindness.^[21,22]^ Therefore, treating corneal neovascularization and haziness is important in the treatment of HSK because the pathophysiology of these factors is due to the presence of inflammatory mediators and T-helper cells.^[23]^ Thus, tacrolimus may target these cells in humans, leading to a significant reduction in corneal pathologies. This was confirmed by the results of the present study. Similar studies have confirmed the efficacy of cyclosporine A in treating HSK-related neovascularization and reducing the number of inflammatory cells and T-lymphocytes,^[9]^ which is consistent with the results of the present study. Additionally, the control group had reduced corneal neovascularization and edema, which is consistent with the results of previous studies that reported the efficacy of antivirals and steroids in reducing neovascularization and edema during HSK.^[8,24,25]^

Corneal epitheliopathy improved in both groups but the improvement was greater in the case group. This confirms the efficacy of tacrolimus for treating corneal epitheliopathy and its efficacy in preventing ulcers during allergic conjunctivitis.^[26]^ However, corneal epitheliopathy has not yet been evaluated for HSK.

The immunity-related mechanism of HSK and ocular pathologies, and the efficacy of tacrolimus in a rat study led us to examine the efficacy of tacrolimus as an immunosuppressant used in treating HSK. Currently available treatments for HSK include antivirals and topical corticosteroids. However, corticosteroid therapy can lead to serious side effects after long-term use. Several clinical pilot studies have attempted to determine the beneficial effects of topical cyclosporin A in the treatment of non-necrotizing HSK, particularly in cases that are not responsive to topical prednisolone.^[27]^ Tacrolimus shares several immunosuppressive properties with cyclosporine A, although it is known to be 10 to 100 times more potent than cyclosporine A. Previous studies on the use of tacrolimus ointment for the treatment of atopic eyelid disease reported a positive response and improvements in conjunctivitis symptoms without adverse events.^[28]^ Corticosteroid therapy in patients with HSK requires a long period of tapered corticosteroid doses because of the consequences associated with the rapid discontinuance of the drug and the accompanying side effects, such as glaucoma, cataracts, infection, or corneal melting. Thus, tacrolimus therapy may represent a novel approach for suppressing HSV-induced corneal immunoreaction and for preventing corneal scarring. In the present study, tacrolimus administration markedly reduced HSK lesion progression and the degree of corneal haziness, edema, and neovascularization.

Taking samples from a single center is a major limitation to the generalizability of the results.

An unavoidable limitation in our study was the criteria used for grading corneal signs. There is currently no validated criteria available for this purpose. Therefore, the method employed in the present study needs to be validated in the future. Another limitation of this study was the relatively short follow-up period, which did not allow for measurements and comparisons of the rate of HSK recurrence in either study groups. There were also several confounders, such as the synergistic effect of the drugs prescribed to the case group. This suggests the need for the comparison of each of these drugs (tacrolimus vs steroids) in future RCTs. In addition, the efficacy of tacrolimus could be compared to those of other immunosuppressive drugs, which have been found to be effective for treating HSK, such as cyclosporine A and topical immunosuppressive drugs without corticosteroids.

In conclusion, in this RCT, the visual acuity of patients and corneal pathologies, such as corneal haziness, edema, neovascularization, and punctate epitheliopathy, significantly improved after the use of 0.05% tacrolimus eye drops four times a day for one month, in addition to acyclovir and prednisolone. Therefore, we suggest that 0.05% tacrolimus eye drops are safe and appropriate for treating HSK, reducing corneal sequels and morbidities, and improving the visual prognosis.

##  Financial Support and Sponsorship

Nil.

##  Conflicts of Interest

There are no conflicts of interest.
